# A Community Study of *Borrelia burgdorferi* Antibodies among Individuals with Prior Lyme Disease in Endemic Areas

**DOI:** 10.3390/healthcare6020069

**Published:** 2018-06-19

**Authors:** Barbara Strobino, Katja Steinhagen, Wolfgang Meyer, Thomas Scheper, Sandra Saschenbrecker, Wolfgang Schlumberger, Winfried Stöcker, Andrea Gaito, Brian A. Fallon

**Affiliations:** 1 Department of Psychiatry, Lyme and Tick-Borne Diseases Research Center, Columbia University Irving Medical Center, New York, NY 10032, USA; baf1@cumc.columbia.edu; 2 Research Foundation for Mental Hygiene, Inc., New York, NY 10032, USA; 3 Institute for Experimental Immunology, Euroimmun, 23560 Lübeck, Germany; k.steinhagen@euroimmun.de (K.S.); w.meyer@euroimmun.de (W.M.); t.scheper@euroimmun.de (T.S.); s.saschenbrecker@euroimmun.de (S.S.); w.schlumberger@euroimmun.de (Wo.S.); w.stoecker@euroimmun.de (Wi.S.); 4 Independent Researcher, Basking Ridge, NJ 07920, USA; adg0339@gmail.com

**Keywords:** Lyme disease, *Borrelia burgdorferi*, serology, ELISA, immunoblot

## Abstract

The objective was to examine the prevalence of *Borrelia* antibodies among symptomatic individuals with recent and past Lyme disease in endemic communities using standard assays and novel assays employing next-generation antigenic substrates. Single- and two-tiered algorithms included different anti-*Borrelia* ELISAs and immunoblots. Antibody prevalence was examined in sera from 32 individuals with recent erythema migrans (EM), 335 individuals with persistent symptoms following treatment for Lyme disease (PTLS), and 41 community controls without a history of Lyme disease. Among convalescent EM cases, sensitivity was highest using the C6 ELISA (93.8%) compared to other single assays; specificity was 92.7% for the C6 ELISA vs. 85.4–97.6% for other assays. The two-tiered ELISA-EUROLINE IgG immunoblot combinations enhanced case detection substantially compared to the respective ELISA-IgG Western blot combinations (75.0% vs. 34.4%) despite similar specificity (95.1% vs. 97.6%, respectively). For PTLS cohorts, two-tier ELISA-IgG-blot positivity ranged from 10.1% to 47.4%, depending upon assay combination, time from initial infection, and clinical history. For controls, the two-tier positivity rate was 0–14.6% across assays. A two-tier algorithm of two-ELISA assays yielded a high positivity rate of 87.5% among convalescent EM cases with specificity of 92.7%. For convalescent EM, combinations of the C6 ELISA with a second-tier ELISA or line blot may provide useful alternatives to WB-based testing algorithms.

## 1. Introduction

Lyme disease, a multisystem disorder caused by tick-transmitted spirochetes of the *Borrelia burgdorferi sensu lato* complex, is widely distributed in the northern hemisphere with an estimated 329,000 cases per year in the United States [1] and an estimated 232,125 cases per year in Europe [2]. After exposure, the majority of patients develop a characteristic rash (erythema migrans, EM) along with nonspecific symptoms. Without adequate antibiotic treatment, infection disseminates within days or weeks, presenting with cardiac, articular, neurologic, skin, and/or ocular manifestations [3]. In a subgroup of patients, symptoms recur or persist for months or years after treatment. These patients are referred to as having post-treatment Lyme disease syndrome (PTLDS) if the emergence of fatigue, pain, or cognitive problems occurred within six months of the original infection [3]. For those individuals for whom the precise onset of infection cannot be determined or whose chronic symptoms may have started more than six months after the initial infection, there is no currently agreed upon syndromal terminology. For the purpose of this paper, we will refer to these individuals as having “post-treatment Lyme symptoms” (PTLS) for distinction from those with the more precise designation PTLDS. 

Diagnosis relies on characteristic clinical symptoms and exposure to ticks in Lyme endemic areas; serologic confirmation is not required for diagnosis in the presence of an EM rash, but is necessary to meet surveillance criteria for later-stage disseminated disease [4,5]. According to recommended guidelines in the United States and Europe, serological diagnosis should follow a two-tiered protocol based on a sensitive first-tier enzyme immunoassay or immunofluorescence assay, followed by confirmatory IgM and IgG immunoblot analysis among those positive or equivocal in the first tier [6,7]. However, there is widespread recognition that these testing strategies have limitations [8,9,10,11,12,13,14,15]. While highly specific, the two-tier strategy is insensitive in the first month of infection. In one recent study of early Lyme disease, only 35.6% of cases tested positive at the time of the rash and convalescent testing 3–4 weeks later added only 25% more cases; this means nearly 40% of the samples tested negative during the first four weeks after the rash using the standard two-tier protocol [16]. Further complicating the picture is that results from the ELISAs and Western blots can vary greatly between laboratories [9,10,17,18,19]. These tests were developed based on *Borrelia* cultures in vitro; this limits assay sensitivity as some antigens (e.g., variable major protein-like sequence expressed (VlsE)) are only expressed in vivo. Variability of antigen expression in the human host based on duration of infection and other factors had not been taken into account. Revision and/or replacement of these tests are clearly needed.

In recent years, recombinant *Borrelia* proteins (such as VlsE) and synthetic peptides (e.g., C6 derived from the immunodominant sixth invariable region of VlsE) have been used as stand-alone assays or added to immunoblot panels that incorporate both native and recombinant or synthetic peptides [12,20,21,22,23,24,25,26,27]. Their use reduces the rate of cross-reactivity, while the combination of multiple antigens expressed at different stages of human infection improves sensitivity. Inclusion of reactivity against newer-generation antigenic substrates such as VlsE was not possible when the 1994 Dearborn consensus criteria for Western blot (WB) interpretation were formulated, but the incorporation of new antigens was recommended in anticipation of their availability [6]. Thus, for the determination of Lyme antibody prevalence in the community setting, comparative analysis by conventional and new assays as well as new algorithms is both timely and informative.

Clinicians from Lyme-endemic areas are often uncertain about how long serologic tests for Lyme disease antibodies remain positive after infection, whether there is a high base rate among those who do not recall ever having had Lyme disease, and to what extent the new developments using recombinant or synthetic peptides impact test sensitivity. To address these questions, the following community serosurvey was conducted in four Lyme-endemic counties in the northeastern United States. In this study, we evaluated symptomatic patients with a history of Lyme disease and community controls without a history of Lyme disease to determine the prevalence of antibodies against *Borrelia* spp. using different diagnostic assays based on native and recombinant peptides, both as single assays and within two-tiered protocols.

## 2. Materials and Methods

### 2.1. Population

In July 2009, serosurveys were conducted in four Lyme disease-endemic counties located in New York, New Jersey, and Connecticut [28]. Advertisements were posted in local papers and distributed by local organizations and health departments. 

Individuals aged 18 to 75 years were enrolled if they met either of the following criteria: (a) presence of an EM rash within 3 months of the screening; or (b) symptoms of fatigue, pain, or cognitive problems attributed to prior treated Lyme disease. Participants completed a questionnaire regarding symptoms associated with their most recent acute episode and previous episodes of Lyme disease, history of tick bites, co-infections, treatments, and blood tests. All subjects gave written informed consent for inclusion before they participated in the study. The study was conducted in accordance with the Declaration of Helsinki, and the protocol was approved by the New York State Psychiatric Institute Institutional Review Board (#5847).

In the 6 months after the screening clinic, we conducted follow-up telephone interviews with selected individuals to confirm their reported histories and to obtain additional clinical data. Criteria for follow-up contact included: (a) report of a new episode of Lyme disease within 3 months prior to the clinic date, or (b) report of persistent symptoms at the time of the clinic following a Lyme disease diagnosis that included CDC surveillance signs of early Lyme disease (EM) or extra-cutaneous disseminated Lyme disease (cranial nerve palsy, heart block, meningitis, radiculoneuropathy, and/or joint swelling) [28] within 5 years of the clinic date. The telephone interview included more detailed questions regarding the EM and confirmation that a clinician saw and diagnosed the EM rash. We successfully contacted 62.2% (79 of 127) who fulfilled the above criteria.

Out of 461 survey participants, 408 were included in the analysis of antibody prevalence. Subjects were excluded because they (i) reported Lyme disease but had not been evaluated or treated at the time by a physician (*n* = 29); (ii) reported an EM, but on review did not fulfill the criteria for EM cases (*n* = 23); or (iii) were unsure of having had Lyme disease (*n* = 1). Case groups were further defined by clinical characteristics and time between initial symptoms and study participation (Table 1).

Case Cohort 1: Early Lyme disease with erythema migrans (EM cases; *n* = 32)

There were 32 individuals who had a classic EM rash (>2 inches) that was either present at the time of the screening or had been diagnosed as an EM rash by a physician within 3 months preceding the screening. Of these 32 cases, 24 reported never having had Lyme disease prior to this occurrence and 8 reported a prior episode of Lyme disease.

Case Cohort 2: Post-treatment Lyme symptoms, confirmed (PTLS-c; *n* = 19)

Based on the initial assessment and follow-up phone interview, this group of individuals fulfilled the CDC surveillance clinical criteria for acute or disseminated Lyme disease >3 months prior to the screening clinic with characteristic signs (e.g., EM rash, cranial nerve palsy, swollen large joint). 

Case Cohorts 3 and 4: Post-treatment Lyme symptoms, presumed (PTLS-p ≤2 years; *n* = 148 and PTLS-p >2 years; *n* = 168)

These individuals with persistent symptoms attributed to Lyme disease reported having been diagnosed or treated for Lyme disease by a physician. Since clinical history was not confirmed through telephone interviews or medical records, these cohorts were presumed to have had an initial acute episode of Lyme disease based on review of the questionnaire. Unlike the PTLS-c group, we did not require that individuals in the PTLS-p groups meet the CDC-defined surveillance clinical case criteria; we simply required that they had been previously diagnosed and treated for Lyme disease. They were further classified as: “≤2 years” if their most recent acute episode of Lyme disease occurred after 1 January 2007; or “>2 years”, if their most recent acute episode of Lyme disease occurred before 1 January 2007.

Control Cohort: Community residents with no history of Lyme disease (*n* = 41)

Less than 10% of individuals came to the clinics for a blood test even though they had never before been diagnosed with Lyme disease. All were concerned about Lyme disease, some of whom had nonspecific symptoms such as joint pain. After reviewing all 461 questionnaires, blinded to the serology results, we identified 41 people who reported never having been diagnosed and/or treated for Lyme disease and whose symptom history was highly inconsistent with a diagnosis of Lyme disease.

### 2.2. Laboratory Tests

**Figure 1:**
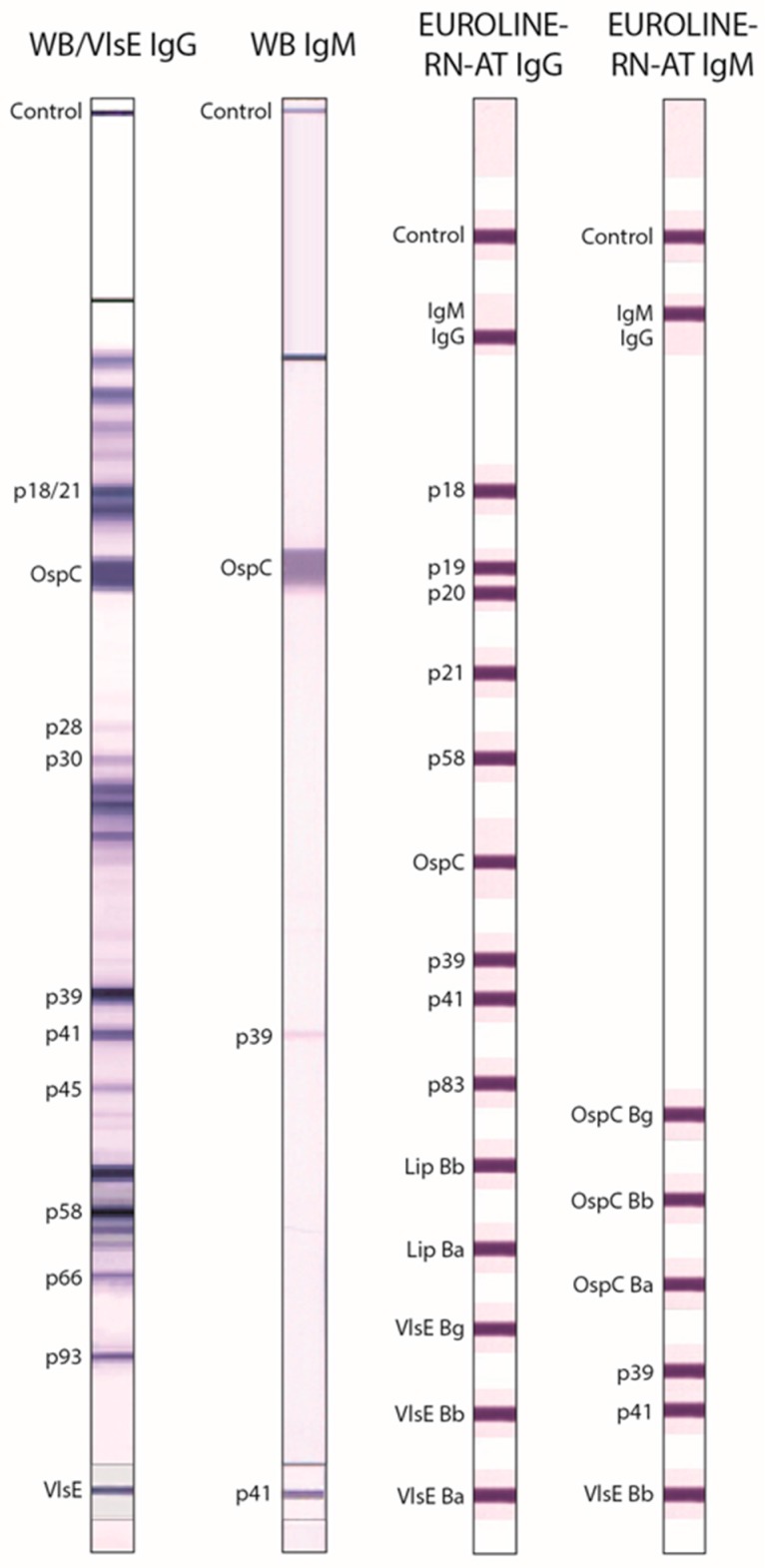
Anti-*Borrelia* immunoblot tests. Representative reactivity of the WB/VlsE IgG and WB IgM after incubation with sera from patients with late Lyme disease (IgG) and erythema migrans (IgM), respectively (left two panels). Schematic representation of antigens used in the line blot assays EUROLINE-RN-AT IgG and EUROLINE-RN-AT IgM (right two panels).

Serological testing was conducted blind to clinical history at the Institute for Experimental Immunology affiliated with Euroimmun, Inc., Lübeck, Germany. Table 2 summarizes the laboratory tests carried out on the samples. Assays were performed according to the manufacturers’ instructions. Immunoblots were evaluated digitally using the EUROLineScan software (Euroimmun). Figure 1 shows an example of a reactive immunoblot. 

Reactivity in the *Borrelia burgdorferi* whole-cell lysate WB was interpreted according to the Dearborn criteria that require reactivity of at least 2 of 3 (IgM) or at least 5 of 10 (IgG) specific bands for a positive result [6]. Reactivity against the supplementary VlsE band in the WB IgG was scored separately since VlsE is not included in the standard Dearborn criteria. The Line immunoblot (EUROLINE-RN-AT) which includes native and recombinant epitopes was interpreted based on the “MiQ 12” criteria that are recommended by the German Society for Hygiene and Microbiology (DGHM). These criteria are commonly used in European laboratories and suggest that a positive immunoblot result is indicated by reactivity of at least 1 of 5 (IgM) or at least 2 of 10 (IgG) specific bands [7]. Because most of the antigens contained in the EUROLINE-RN-AT are derived from *Borrelia burgdorferi* B31, which is the most relevant strain for the United States, this assay was considered both interesting and suitable for analysis of the study cohorts. The C6 ELISA and the WB IgG have been approved for in vitro diagnostics by the United States Food and Drug Administration (FDA).

### 2.3. Statistical Methods

McNemar’s chi-square test was used to compare assays. *p*-values < 0.05 were considered significant. Confidence limits of proportions were calculated at the 95% confidence level using the binomial exact method. All analyses were carried out with SPSS, ver. 23 (SPSS Inc., Chicago, IL, USA).

## 3. Results

### 3.1. Cohort Characteristics

There were no significant age and gender differences between the case and control cohorts (Table 3). Participants were asked if they had previously had a positive Lyme test. Although the specific test (ELISA, Western blot, PCR) was not known in the majority of cases, approximately half of each of the PTLS cohorts reported having had a positive Lyme test previously, in contrast to 28% of the EM case group. PTLS-p cohorts were 7–8 times more likely than controls to report having had another tick-borne disease. Tick bites within one year of the study were common in all cohorts. Among the EM cases, 75% recalled at least one tick bite within one year of the study. 

By definition, all of the EM cases presented with a rash, with over two-thirds reporting accompanying joint pain, fatigue, and headache. Joint pain and fatigue were the most common symptoms reported in all PTLS cohorts, followed by mental confusion or fog. 

The prevalence of C6 positives among all participants did not differ significantly by county: 21.3% in Fairfield County, CT; 29.3% in Somerset County, NJ; 31.6% in Dutchess County, NY, USA; and 33.0% in Columbia County, NY, USA (*p* = 0.51). 

### 3.2. Antibody Prevalence: Single-Tier Testing

Using single-tier testing, the prevalence of antibodies against *Borrelia* spp. declined from up to 100% among individuals tested in the first month post symptom onset to, on average, less than 20% among those in later disease stages (PTLS-p >2 years) (Table 4). 

Among the EM cohort, the highest percentage of positive samples (93.8%) derived from a single assay was observed with the C6 ELISA. Positivity rates by Euroimmun ELISAs were lower for separate IgG (81.3%) and IgM (75.0%) testing, while the result of combined IgG and IgM analysis (87.5%) was not significantly different from the C6 ELISA. In EM cases with symptoms of ≤30 days’ duration, antibody prevalence was higher than that among cases with >30 days from EM onset, reaching 100% on several tests (C6 ELISA, anti-VlsE ELISA IgG or anti-*Borrelia* ELISA IgG plus VlsE/IgM). 

In all PTLS cohorts, the anti-*Borrelia* ELISA IgG/IgM was significantly more likely to be positive than the C6 ELISA. This higher sensitivity for the anti-*Borrelia* ELISA is not surprising given the differences in specificity between the two tests (85.4% for the anti-*Borrelia* ELISA IgG plus VlsE/IgM vs. 92.7% for the C6 ELISA); this difference however was not statistically significant. The differences between assays persist even if only those PTLS cases with negative or inconclusive test results in the past are considered (*n* = 68), although the rates of positivity in this group are lower than those observed among PTLS cases with at least one previous positive result (*n* = 167). For example, 41.3% of those with a previous positive test result tested positive by anti-*Borrelia* plus VlsE ELISA IgG, and 25% of those PTLS cases with only negative or inconclusive past test results were positive by anti-*Borrelia* plus VlsE ELISA IgG. Positivity for C6 ELISA was 33.5% and 16.2%, respectively.

Highest IgG positivity rates among immunoblots were obtained by the EUROLINE-RN-AT IgG, most evident in the EM group. Here, sensitivity by WB IgG was only 34.4% compared to 75.0% by EUROLINE-RN-AT IgG; specificity was slightly greater for WB IgG than EUROLINE-RN-AT IgG (97.6% vs. 92.7%). Notably, the VlsE band accounted for a large proportion of the increased detection rate; when the recombinant VlsE band alone was assessed on an immunoblot, the IgG positivity rate amounted to 68.8%. In all study cohorts, the EUROLINE-RN-AT IgM produced significantly more positive results than the WB IgM. 

### 3.3. Antibody Prevalence: Two-Tiered Testing Using ELISA–Immunoblot Combinations

Two-tiered approaches were evaluated with C6 ELISA or anti-*Borrelia* ELISA IgG plus VlsE/IgM as first tier and different immunoblots as the second tier (Table 5). A positive test result was defined as a positive/equivocal first-tier ELISA and a positive second-tier immunoblot. 

Among early EM cases (≤30 days), using the C6 ELISA as the first-tier test, the prevalence of positive results by EUROLINE-RN-AT IgM blot as the second-tier test was substantially, albeit not significantly, higher than by the analogous WB IgM (93.8% vs. 62.5%), but with a slight reduction in specificity (97.6% vs. 100%). Similarly, among these early cases, using the C6 ELISA as the first-tier test, the positivity rate determined by EUROLINE-RN-AT IgG blot as the second-tier test was substantially higher than that by the analogous WB IgG (87.5% vs. 37.5%, *p* < 0.05). Among late EM cases (>30 days), using the C6 ELISA or anti-*Borrelia* ELISA IgG plus VlsE/IgM as the first-tier test, the use of the EUROLINE-RN-AT IgG immunoblot led to the detection of twice as many cases as with the WB IgG (62.5% vs. 31.3%) with only a minimal difference in specificity (95.1% vs. 97.6%). Notably, these findings in the EM cases are sustained even when those individuals with a past history of Lyme disease are removed, indicating that the results are not confounded by previous infection. 

For the PTLS cohorts, the differences between positivity rates using the EUROLINE-RN-AT IgG versus WB IgG as the second-tier immunoblot were negligible. 

### 3.4. Antibody Prevalence: Two-Tiered Testing Using Two-ELISA Combinations

Among EM cases, the combination of the C6 ELISA followed by the anti-*Borrelia* ELISA IgG plus VlsE/IgM or the inverse algorithm (anti-*Borrelia* ELISA IgG plus VlsE/IgM followed by the C6 ELISA) demonstrated the highest positivity rates of 100% (≤30 days) and 75.0% (>30 days), at a specificity of 92.7% (Table 6). Two-tier testing using these two-ELISA combinations yielded the same positivity rate (EM cases) and specificity (controls) as the two-tier testing using the ELISA-immunoblot algorithm (C6-ELISA and the EUROLINE-RN-AT IgG/IgM). In addition, the two-tier ELISA algorithm significantly outperformed all of the standard two-tier ELISA and WB approaches in ability to detect seropositivity. 

## 4. Discussion

This study demonstrates that serologic tests that incorporate recombinant antigens (e.g., VlsE) or synthetic peptides (e.g., C6) had a high detection rate of *Borrelia* antibodies in the early convalescent phase after EM (93.8%) with a specificity of 92.7%. This contrasts favorably with results in a report by Rebman et al. [16] of 104 patients with EM which indicated that 79.8% had positive serologic test results using a sonicated whole-cell *B. burgdorferi* ELISA and 60.6% had positive test results using the two-tier algorithm combining the whole-cell *B. burgdorferi* ELISA and Western blot (either at initial presentation or at convalescent testing 3–4 weeks after doxycycline treatment). Clinicians in Lyme-endemic areas often prefer assays that have the highest sensitivity so as to reduce the risk of missing a true case of Lyme disease; while this may not be optimal for epidemiologic surveillance, in which specificity is of primary importance, it may be best for clinical decision making in Lyme-endemic areas, where the greater harm might be seen as not treating someone who is truly infected. Diagnostic guideline developers, aiming for higher levels of specificity, use a two-tier algorithm to achieve fewer false positives. 

Many physicians in the United States may not be aware of tests that incorporate VlsE or C6 that are currently on the market. The C6 ELISA—the first synthetic peptide assay for Lyme disease in the United States—has been on the market for over a decade, but still is not widely used, despite its superior sensitivity and specificity compared to the standard whole-cell sonicate ELISA. 

When a two-tier algorithm of the C6 ELISA with the IgG or IgM WB was used, the positivity rate in convalescent EM dropped from 93.8% for the C6 as a single assay to 56.3% for the C6–IgM combination and to 34.4% for the C6–IgG combination. While the specificity rate among controls increased slightly from 92.7% to 97.6%, most clinicians would consider this to be an unacceptably high price to pay in loss of sensitivity for a small increase in specificity. The next generation immunoblots employed in this study improve upon the WB. When the C6 ELISA was combined with the EUROLINE IgG or IgM blot in the two-tier protocol, sensitivity remained high (75–78.1%) while specificity was slightly enhanced (95.1–97.6%). Both combinations enhance case detection in convalescent EM by 22–40% compared to the respective C6 ELISA and WB assay, with statistically insignificant differences in the specificity among controls. When we examined the key driver of enhanced detection in these assays, it was clearly the inclusion of the VlsE protein, indicating that assays and interpretive criteria should incorporate VlsE or derivatives thereof. 

Another criticism of the WB has been that it is time-consuming and costly. A two-tier ELISA algorithm would be faster and less expensive than conventional two-tiered (ELISA–immunoblot) algorithms. As seen in this study, the combination of the C6 ELISA and the anti-*Borrelia* ELISA IgG plus VlsE/IgM outperformed ELISA–WB approaches; a finding consistent with other studies [13,29,30,31]. 

Among individuals from a Lyme-endemic area who do not have a history of Lyme disease who came to be screened, the base rate of positivity for a single ELISA assay ranged from 2.4% to 14.6%; the most specific assays were ELISAs that included C6 or VlsE, yielding specificities from 92.7% to 97.6%. Clinicians therefore should consider replacing the first-tier whole-sonicate ELISA with these more specific ELISAs. 

The specificity for both single- and two-tiered assays protocols in this study is likely to be an underestimate of the actual rate. In earlier studies, specificity rates for two-tier algorithms ranged between 97% and 100% with U.S. or European immunoblots as the second tier [12,13,14,21,32,33]. Our community control sample included individuals who felt healthy but just wanted to be tested as well as those who were concerned about possibly having Lyme disease based on nonspecific symptoms. While none of the controls reported having ever been diagnosed or treated for Lyme disease, over one-third did recall prior tick bites. Thus, some of our controls may have had unrecognized and untreated prior *Borrelia* infection. Indeed, one of the three controls who tested positive on the C6 ELISA also tested positive on the WB IgG and EUROLINE-RN-AT IgG; this individual most likely did have prior infection with *B. burgdorferi*. When removed post hoc from the control cohort, the specificity on the stand-alone tests improved to 95% for the C6 ELISA and 100% for the WB IgG. 

Assay performance can differ widely from lab to lab [17], and the choice of ELISA–immunoblot combination can markedly influence the number of positive results [10]. As demonstrated here and by others [34], EUROLINE-RN-AT assays exceed the detection rate of WBs in early convalescent Lyme disease. Possible explanations include differences in the interpretive criteria applied for each particular assay [6,7] and the addition of the recombinant VlsE, which is a diagnostically relevant protein not expressed by *Borrelia* cultured in vitro. The use of recombinant VlsE has been shown previously to improve sensitivity of IgG detection without substantial loss of specificity [12,22,23]. In the present study, VlsE IgG reactivity (extra VlsE band coated on the WB IgG) doubled the number of positive IgG results obtained by standard WB, confirming the indispensability of VlsE (or VlsE-derived antigens) in serology-based diagnosis.

Across all assays, we observed an overall decline in assay positivity with time, ranging from up to 100% in the first month after symptom onset to, on average, less than 20% years later. These results confirm that antibody positivity among patients with previously treated Lyme disease is common and can remain positive for years. This finding, while not surprising given immunologic memory, highlights the limitations of antibody-based tests and poses a challenge for physicians when evaluating patients with persistent previously treated symptoms. New approaches move beyond the antibody-based assays and offer the promise of addressing the clinically important question of whether or not infection is still present in a previously treated patient. Two have recently been reported, including one that uses nanotechnology to detect a small amount of *Borrelia* antigen [35] and another that uses a T-cell stimulation paradigm to measure IFN-γ production after *Borrelia* antigen challenge [36].

This decline in detection of *Borrelia* antibodies over time in most cases reflects the natural decline in *Borrelia*-specific antibody production after the initial infection. We cannot however exclude the possibility that the negative antibody test results in this study in some patients reflect the insensitivity of the assays in later phases of Lyme disease, either due to low levels of persistent infection or persister *Borrelia* that express different outer surface proteins for which novel assays are required. Studies in rhesus macaques, for example, have demonstrated a decline in C6 antibodies even among macaques with confirmed persistent *Borrelia burgdorferi* infection [37]. This group also demonstrated that Lyme disease tests incorporating a set of antigens that reflect both early and later stages of Lyme disease enhance sensitivity of detection of *Borrelia burgdorferi* antibodies [27].

There are several limitations of the study. First, this was not a random sampling epidemiologic study, but rather a study of symptomatic patients who responded to advertisements in local media. Therefore, we cannot conclude that our seropositivity rates represent the full cohort of previously treated symptomatic individuals in a Lyme-endemic area. Second, this was not a study of early EM prior to treatment, but rather of individuals who were undergoing active treatment or had recently completed antibiotic treatment. Hence, to compare our serologic results to those from other studies, the focus should be on results from convalescent samples rather than on pretreatment cases. The two-tier assay sensitivity for the C6 ELISA and WB IgM combination (62.5%) among individuals tested at ≤4 weeks in our study was comparable to that reported by Rebman et al. [16] using the whole-cell sonicate ELISA and WB IgM (60.6%). Third, our study design was cross-sectional, identifying patients at one point in time, relying upon patient report for diagnosis. Although our results are comparable to others, a prospective design would have been preferable to ensure accuracy of diagnosis. Fourth, as noted previously, the use of individuals as community controls from a Lyme-endemic area may well have biased our study against an accurate representation of the assays’ true specificity. Fifth, while we are most confident of diagnostic accuracy within the convalescent EM and PTLS-confirmed groups, we are also aware that some individuals in the PTLS-presumed groups may never have had prior Lyme disease, partly because the diagnosis and treatment of Lyme-like symptoms in Lyme-endemic areas is sometimes based on probability rather than certitude. Additionally, we are aware that a past positive serology increased the likelihood of a diagnosis of Lyme disease in the PTLS cases; thus, there is a bias towards a positive serology for the study assays in PTLS cases. However, one-quarter of PTLS cases who had never tested positive with standard ELISA or Western blot protocols were positive by anti-*Borrelia* plus VlsE ELISA IgG. While this could reflect a greater sensitivity of the study assay compared to the patient’s previous assay, the positivity could also reflect a previously undetected infection after a new tick bite. Finally, although our sample size of probable PTLS patients was large, representing a strength of this study, the sample size of early EM cases was small, resulting in insufficient power to detect moderate differences among assays. Given a positivity rate of 93.8% for the C6 ELISA as a single-tier test, a comparison test had to have classified 25% of EM cases as negative to be considered significantly different (alpha = 0.05, power = 0.80, two-tailed) in this population. Thus, we recommend that the intriguing results from this study be confirmed in larger studies using prospectively collected samples starting from initial EM presentation.

## 5. Conclusions

In summary, this study provides a representative characterization of Lyme disease seroprevalence among symptomatic individuals with prior Lyme disease from highly endemic areas. This study demonstrates that antibody positivity rates across all tests decline with time from the initial infection and that in a highly endemic area, there is a low base rate of positivity among community controls who do not have a history of prior Lyme disease. This study also conveys encouraging news: that newer diagnostic assays that include recombinant or synthetic peptides or use different testing combination algorithms improve test sensitivity. Strikingly, incorporation of the VlsE antigen (or a derivative thereof) into an assay is indispensable to increase the serological hit rate. This study also demonstrates the value of the C6 ELISA as a stand-alone test and the greater sensitivity in convalescent Lyme disease of using a next-generation immunoblot in two-tier algorithms. Finally, this study confirms that a two-tier ELISA performs as well as the ELISA-line blot combination, thus decreasing cost and increasing efficiency. As an aid to evaluation and treatment of individuals in Lyme-endemic areas with early Lyme disease, these novel assays and algorithms provide new more clinically useful diagnostic tools to the primary care clinician. 

## Figures and Tables

**Table 1:** Study cohort criteria in Lyme-endemic communities, July 2009.

Study Cohort	N	Symptoms at Time of Screening	Reported Past Clinical History from Questionnaire Review and/or Telephone Interview
(1) EM cases (convalescent LD with EM)	32	Observed EM rash (≥2 inches) or physician diagnosed EM rash within 3 months of screening; time from symptom onset ≤30 days (*n* = 16) or >30 days (*n* = 16)	No earlier LD (*n* = 24); prior LD (*n* = 8); verified through phone follow-up
(2) PTLS-confirmed (PTLS-c)	19	Persistent Lyme symptoms of fatigue, pain, or cognitive problems	Diagnosed and treated for EM > 3 months ago and/or diagnosed with disseminated extra-cutaneous LD ^a^; verified through questionnaire review or phone follow-up
(3) PTLS-presumed (PTLS-p) ≤2 years	148	Persistent Lyme symptoms of fatigue, pain, or cognitive problems	Diagnosed and treated for LD after 2007 (most recent acute episode ≤2 years before screening)
(4) PTLS-presumed (PTLS-p) >2 years	168	Persistent Lyme symptoms of fatigue, pain, or cognitive problems	Diagnosed and treated for LD before 2007 (most recent acute episode >2 years before screening)
(5) Community controls ^b^	41	Nonspecific symptoms (e.g., arthralgias, myalgias, fatigue)	No Lyme history (never diagnosed or treated for LD)

^a^ According to the CDC surveillance clinical criteria for Lyme disease [28]. ^b^ Each clinical control case was reviewed by the study physician (BAF) to confirm that the clinical history was highly inconsistent with LD. EM, erythema migrans; LD, Lyme disease; PTLS, post-treatment Lyme symptoms.

**Table 2:** Diagnostic assays evaluated in community screening survey, July 2009.

Assay	Full Test Name (Manufacturer)	Ig Class	Antigen	Interpretation
ELISA				
C6 ELISA	C6 Bb Lyme ELISA (Immunetics, Boston, MA, USA)	IgG, IgM	Synthetic C6 peptide (25 aa) derived from IR_6_ of VlsE Bb (strain B31)	Index: ≤0.90 negative, 0.91–1.09 equivocal, ≥1.10 positive.
Anti-*Borrelia* plus VlsE ELISA IgG	Anti-*Borrelia* plus VlsE ELISA IgG (Euroimmun, Lübeck, Germany)	IgG	Mix of whole-cell antigen extracts from Bb, Ba and Bg plus VlsE Bb *	RU/mL: <16 negative, ≥16 to <22 equivocal, ≥22 positive.
Anti-VlsE ELISA IgG	Anti-Bb VlsE ELISA IgG (Euroimmun)	IgG	VlsE Bb *	RU/mL: <16 negative, ≥16 to <22 equivocal, ≥22 positive.
Anti-*Borrelia* ELISA IgM	Anti-*Borrelia* ELISA IgM (Euroimmun)	IgM	Mix of whole-cell antigen extracts from Bb, Ba and Bg	RU/mL: <16 negative, ≥16 to <22 equivocal, ≥22 positive.
Immunoblot				
WB IgG **	US Anti-Bb EUROLINE-WB IgG (Euroimmun)	IgG	Whole-cell antigen extract from Bb	Positive if ≥5 of the following 10 bands were present: 18/21 kDa, OspC, 28 kDa, 30 kDa, 39 kDa (BmpA), 41 kDa (Fla), 45 kDa, 58 kDa, 66 kDa, 93 kDa; according to Dearborn criteria [6].
VlsE IgG **		IgG	VlsE Bb *	Only the VlsE band was scored. Positive if the VlsE band was present.
WB IgM	US Anti-Bb EUROLINE-WB IgM (Euroimmun)	IgM	Whole-cell antigen extract from Bb plus p41 *	Positive if ≥2 of the following 3 bands were present: OspC, 39 kDa (BmpA), 41 kDa (Fla); according to Dearborn criteria [6].
EUROLINE-RN-AT IgG	Anti-*Borrelia* EUROLINE-RN-AT IgG (Euroimmun)	IgG	p18 *, p19 *, p20 *, p21 *, p58 *, OspC *, p39 *, p41 *, p83 *, Lipid Bb, Lipid Ba, VlsE Bg *, VlsE Bb *, VlsE Ba *	Positive if ≥2 of the following 10 bands were present: p18, p19, p20, p21, p58, OspC, p39, p83, Lipid Bb, and Lipid Ba; or if ≥1 VlsE band was present even if no other specific bands were positive; according to European MiQ 12 criteria [7].
EUROLINE-RN-AT IgM	Anti-*Borrelia* EUROLINE-RN-AT IgM (Euroimmun)	IgM	OspC Bg, OspC Bb, OspC Ba, p39 *, p41 *, VlsE Bb *	Positive if ≥1 of the following 5 bands was present: OspC Bg, OspC Bb, OspC Ba, p39, VlsE Bb; according to European MiQ 12 criteria [7].

* Purified recombinant protein. ** WB IgG test strips were each fitted with a membrane chip coated with recombinant VlsE antigen (Figure 1). Because reactivity against VlsE is not included in the Dearborn criteria, the VlsE band was evaluated separately (VlsE IgG). aa, amino acids; Bb, *Borrelia burgdorferi* sensu stricto; Ba, *Borrelia afzelii*; Bg, *Borrelia garinii*; BmpA, *Borrelia* membrane protein A; ELISA, enzyme-linked immunosorbent assay; Fla, Flagellin; IgG, immunoglobulin G; IgM, immunoglobulin M; IR_6_, sixth invariable region of VlsE; OspC, outer surface protein C; VlsE, variable major protein-like sequence, expressed; WB, Western blot.

**Table 3:** Cohort characteristics and reported symptoms of case cohorts in Lyme-endemic communities, July 2009.

	EM Cases (*n* = 32)	PTLS-c (*n* = 19)	PTLS-p ≤2 Years (*n* = 148)	PTLS-p >2 Years (*n* = 168)	Community Controls (*n* = 41)
**Mean age (±SD)**	56.3 (±12.9)	57.8 (±9.7)	53.5 (±13.1)	56.0 (±12.4)	57.0 (±10.4)
**Gender (% male)**	50.0	42.1	40.5	34.5	51.2
**% Reporting**					
Past positive Lyme test	28.1 **	57.9 ***	54.7 ***	44.6 ***	0.0
Past other tick-borne disease	3.1	5.3	19.6 *	16.7 *	2.4
≥1 tick bite in last year	75.0 *	31.6	54.1	32.7	41.5
**Symptoms of recent Lyme disease episode from survey questionnaire (% with symptoms)**					
Joint pain	71.9	84.2	87.2	81.5	
Fatigue	71.9	78.9	87.2	85.7	
Headache	71.9	47.4	59.5	62.5	
Stiff neck	53.1	57.9	63.5	60.7	
Shooting/stabbing pains	21.9	31.6	33.1	41.1	
Mental confusion/fog	40.6	78.9	60.1	64.3	
Erythema migrans-like rash	100.0	68.4	42.6	48.8	
Joint swelling	31.3	42.1	40.5	38.1	
Light/sound sensitive	18.8	26.3	28.4	34.5	
Fever > 100 °F	46.9	42.1	21.6	27.4	
Cranial nerve palsy	6.3	31.6	2.7	6.5	
Heart block	0	5.3	2.7	1.2	

* *p* < 0.05, ** *p* < 0.01, *** *p* < 0.001; chi-square with Yates correction for continuity, community controls as reference group.

**Table 4:** Prevalence of anti-*Borrelia* antibodies by single-tier testing among patients with erythema migrans or post-treatment Lyme symptoms (PTLS).

Assay	% Positive (95% Confidence Limits)						% Negative (95% Confidence Limits)
	Erythema Migrans			PTLS-c (*n* = 19)	PTLS-p ≤2 Years (*n* = 148)	PTLS-p >2 Years (*n* = 168)	Community Controls (*n* = 41)
	All (*n* = 32)	≤30 Days ^a^ (*n* = 16)	>30 Days ^a^ (*n* = 16)				
ELISA							
C6 ELISA	93.8 (79.2, 99.2)	100.0 (79.4, 100.0)	87.5 (61.7, 98.4)	52.6 (28.9, 75.6)	34.5 (26.8, 42.7)	17.3 (11.9, 23.8)	92.7 (80.1, 98.5)
Anti-*Borrelia* ELISA IgG plus VlsE/IgM ^b^	87.5 (71.0, 96.5)	100.0 (79.4, 100.0)	75.0 (47.6, 92.7)	78.9 * (54.4, 93.9)	50.0 *** (41.7, 58.3)	38.7 *** (31.3, 46.5)	85.4 (70.8, 94.4)
Anti-*Borrelia* plus VlsE ELISA IgG	81.3 (63.6, 92.8)	93.8 (69.8, 99.8)	68.8 (41.3, 89.0)	63.2 (38.4, 83.7)	37.8 (30.0, 46.2)	25.0 ** (18.7, 32.3)	92.7 (80.1, 98.5)
Anti-VlsE ELISA IgG	81.3 (63.6, 92.8)	100.0 (79.4, 100.0)	62.5 (35.4, 84.8)	47.4 (24.4, 71.1)	31.8 (24.4, 39.9)	14.3 (9.4, 20.5)	97.6 (87.1, 99.9)
Anti-*Borrelia* ELISA IgM	75.0 * (56.6, 88.5)	81.3 (54.4, 96.0)	68.8 (41.3, 89.0)	47.4 (24.4, 71.1)	30.4 (23.1, 38.5)	26.2 * (19.7, 33.5)	92.7 (80.1, 98.5)
Immunoblot							
WB IgG	34.4 *** (18.6, 53.2)	37.5 ** (15.2, 64.6)	31.3 (11.0, 58.7)	42.1 (20.3, 66.5)	25.7 (18.9, 33.5)	13.1 (8.4, 19.2)	97.6 (87.1, 99.9)
VlsE IgG	68.8 (50.0, 83.9)	81.3 (54.4, 96.0)	56.3 (29.9, 80.2)	42.1 (20.3, 66.5)	25.7 (18.9, 33.5)	11.9 (7.4, 17.8)	97.6 (87.1, 99.9)
WB IgM	56.3 * (37.7, 73.6)	62.5 (35.4, 84.8)	50.0 (24.7, 75.3)	0.0 * (0.0, 17.6)	7.4 *** (3.8, 12.9)	0.6 *** (0, 3.3)	97.6 (87.1, 99.9)
EUROLINE-RN-AT IgG	75.0 (56.6, 88.5)	87.5 (61.7, 98.4)	62.5 (35.4, 84.8)	47.4 (24.4, 71.1)	29.1 (21.9, 37.1)	15.5 (10.4, 21.8)	92.7 (80.1, 98.5)
EUROLINE-RN-AT IgM	78.1 (60.0, 90.7)	93.8 (69.8, 99.8)	62.5 (35.4, 84.8)	26.3 (9.1, 51.2)	23.0 (16.5, 30.6)	15.5 (10.4, 21.8)	90.2 (76.9, 97.3)

* *p* < 0.05, ** *p* < 0.01, *** *p* < 0.001 (McNemar’s chi-square with C6 as contrast reference for ELISAs and EUROLINE-RN-AT IgG or IgM as reference for US WB). ^a^ Number of days from onset of erythema migrans to test. ^b^ Combined evaluation of the anti-*Borrelia* plus VlsE ELISA IgG and the anti-*Borrelia* ELISA IgM.

**Table 5:** Prevalence of anti-*Borrelia* antibodies by two-tiered testing (ELISA-immunoblot) among patients with erythema migrans or post-treatment Lyme symptoms (PTLS).

First-Tier ELISA	Second-Tier Immunoblot ^a^	% Positive (95% Confidence Limits) ^b^						% Negative (95% Confidence Limits) ^c^
		Erythema Migrans			PTLS-c (*n* = 19)	PTLS-p ≤2 Years (*n* = 148)	PTLS-p >2 Years (*n* = 168)	Community Controls (*n* = 41)
		All (*n* = 32)	≤30 Days ^d^ (*n* = 16)	>30 Days ^d^ (*n* = 16)				
C6 ELISA	WB IgG	34.4 *** (18.6, 53.2)	37.5 ** (15.2, 64.6)	31.3 (11.0, 58.7)	36.8 (16.3, 61.6)	23.0 (16.5, 30.6)	10.1 (6.0, 15.7)	97.6 (87.1, 99.9)
	VlsE IgG	68.8 (50.0, 83.9)	81.3 (54.4, 96.0)	56.3 (29.9, 80.2)	42.1 (20.3, 66.5)	25.0 (18.3, 32.8)	11.3 (6.9, 17.1)	97.6 (87.1, 99.9)
	WB IgM	56.3 * (37.7, 73.6)	62.5 (35.4, 84.8)	50.0 (24.7, 75.3)	0.0 (0, 17.6)	7.4 *** (3.8, 12.9)	0.0 *** (0.0, 2.2)	100 (91.4, 100.0)
	WB IgG/IgM ^e^	78.1 (60.0, 90.7)	87.5 (61.7, 98.4)	68.8 (41.3, 89.0)	36.8 (16.3, 61.6)	24.3 * (17.7, 32.1)	10.1 (6.0, 15.7)	97.6 (87.1, 99.9)
	EUROLINE-RN-AT IgG	75.0 (56.6, 88.5)	87.5 (61.7, 98.4)	62.5 (35.4, 84.8)	36.8 (16.3, 61.6)	27.0 (20.1, 34.9)	13.1 (8.4, 19.2)	95.1 (83.5, 99.4)
	EUROLINE-RN-AT IgM	78.1 (60.0, 90.7)	93.8 (69.8, 99.8)	62.5 (35.4, 84.8)	15.8 (3.4, 39.6)	16.9 (11.2, 23.9)	7.1 (3.7, 12.1)	97.6 (87.1, 99.9)
	EUROLINE-RN-AT IgG/IgM ^e^	87.5 (71.0, 96.5)	100.0 (79.4, 100.0)	75.0 (47.6, 92.7)	42.1 (20.3, 66.5)	29.1 (21.9, 37.1)	13.7 (8.9, 19.8)	92.7 (80.1, 98.5)
Anti-*Borrelia* ELISA IgG plus VlsE/IgM ^f^	WB IgG	34.4 *** (18.6, 53.2)	37.5 ** (15.2, 64.6)	31.3 (11.0, 58.7)	42.1 (20.3, 66.5)	25.7 (18.9, 33.5)	13.1 (8.4, 19.2)	97.6 (87.1, 99.9)
	VlsE IgG	68.8 (50.0, 83.9)	81.3 (54.4, 96.0)	56.3 (29.9, 80.2)	42.1 (20.3, 66.5)	25.7 (18.9, 33.5)	11.9 (7.4, 17.8)	97.6 (87.1, 99.9)
	WB IgM	56.3 * (37.7, 73.6)	62.5 (35.4, 84.8)	50.0 (24.7, 75.3)	0.0 * (0, 17.6)	7.4 *** (3.8, 12.9)	0.6 *** (0, 3.3)	97.6 (87.1, 99.9)
	WB IgG/IgM ^e^	78.1 (60.0, 90.7)	87.5 (61.7, 98.4)	68.8 (41.3, 89.0)	42.1 (20.3, 66.5)	27.0 ** (20.1, 34.9)	13.7 ** (8.9, 19.8)	95.1 (83.5, 99.4)
	EUROLINE-RN-AT IgG	75.0 (56.6, 88.5)	87.5 (61.7, 98.4)	62.5 (35.4, 84.8)	47.4 (24.4, 71.1)	28.4 (21.3, 36.4)	14.3 (9.4, 20.5)	95.1 (83.5, 99.4)
	EUROLINE-RN-AT IgM	78.1 (60.0, 90.7)	93.8 (69.8, 99.8)	62.5 (35.4, 84.8)	26.3 (9.1, 51.2)	22.3 (15.9, 29.9)	14.3 (9.4, 20.5)	90.2 (76.9, 97.3)
	EUROLINE-RN-AT IgG/IgM ^e^	87.5 (71.0, 96.5)	100.0 (79.4, 100.0)	75.0 (47.6, 92.7)	57.9 (33.5, 79.7)	35.8 (28.1, 44.1)	22.0 (16.0, 29.1)	85.4 (70.8, 94.4)

* *p* < 0.05, ** *p* < 0.01, *** *p* < 0.001 (McNemar’s chi-square with EUROLINE-RN-AT approach as reference for US WB approach). ^a^ Immunoblot used for second test with a positive or equivocal ELISA. ^b^ A positive test result was defined as a positive/equivocal first-tier ELISA and a positive second-tier immunoblot. ^c^ Negativity was defined as a negative result in either or both tiers. ^d^ Number of days from onset of erythema migrans to test. ^e^ Combined evaluation of IgG and IgM immunoblot assay. ^f^ Combined evaluation of the anti-*Borrelia* plus VlsE ELISA IgG and the anti-*Borrelia* ELISA IgM.

**Table 6:** Prevalence of anti-*Borrelia* antibodies by two-tiered testing (ELISA–ELISA) among patients with erythema migrans.

First-Tier ELISA	Second-Tier ELISA	% Postive (95% Confidence Limits)			% Negative (95% Confidence Limits)
		Erythema Migrans			Community Controls (*n* = 41)
		All (*n* = 32)	≤30 Days ^a^ (*n* = 16)	>30 Days ^a^ (*n* = 16)	
C6 ELISA	Anti-*Borrelia* ELISA IgG plus VlsE/IgM ^b^	87.5 (71.0, 96.5)	100.0 (79.4, 100.0)	75.0 (47.6, 92.7)	92.7 (80.1, 98.5)
	Anti-*Borrelia* plus VlsE ELISA IgG	81.3 (63.6, 92.8)	93.8 (69.8, 99.8)	68.8 (41.3, 89.0)	95.1 (83.5, 99.4)
	Anti-VlsE ELISA IgG	81.3 (63.6, 92.8)	100.0 (79.4, 100.0)	62.5 (35.4, 84.8)	97.6 (87.1, 99.9)
	Anti-*Borrelia* ELISA IgM	75.0 (56.6, 88.5)	81.3 (54.4, 96.0)	68.8 (41.3, 89.0)	97.6 (87.1, 99.9)
Anti-*Borrelia* ELISA IgG plus VlsE/IgM ^b^	C6 ELISA	87.5 (71.0, 96.5)	100.0 (79.4, 100.0)	75.0 (47.6, 92.7)	92.7 (80.1, 98.5)

^a^ Number of days from onset of erythema migrans to test. ^b^ Combined evaluation of the anti-*Borrelia* plus VlsE ELISA IgG and the anti-*Borrelia* ELISA IgM.
